# Letter from W. P. Blake, New York

**Published:** 1848-04

**Authors:** W. P. Blake

**Affiliations:** New York


### ARTICLE VIII.

### *Letter from* W. P. Blake, *New York*.

Dear Sir:—I think it desirable to communicate to you, for publication in the Journal, an important application of the new material, gutta percha, to dental prosthesis.

While experimenting with this substance, recently, its adaptation to procuring exact impressions of the teeth and gums occurred to me, and was tested with very satisfactory results. Its great plasticity and softness, when heated above 100 deg., permits it to take the form of the teeth and gums as readily and perfectly as wax; and when, in a few moments, its temperature has lowered, it resumes its tough and elastic state, and, consequently, may be withdrawn without the least change or injury; having no "drags," as they are technically termed; this will give a plaster cast with the necks of the teeth perfectly formed, which cannot be obtained from wax or other inelastic material.

To prepare it for use, the purified and prepared gum is placed in hot water, until sufficiently soft to be moulded into the form required and pressed into the frame, which may be "undercut" in order to hold it more securely; the whole may be repeatedly immersed in the water until it is ready for the mouth; after being pressed sufficiently upon the gums, it must be carefully retained there from two to three minutes until it becomes hardened. To relieve the plaster cast, it is only necessary to warm the gutta percha by water, or otherwise to insure its safe delivery. The crowns of the teeth on the cast may be trimmed at discretion, to facilitate their drawing from sand, but their necks need no alteration. Close and perfect contact of clasps can, therefore, be at once attained, saving much time and labor in adjusting to the mouth.

There are many other purposes for which this singular substance can be used with advantage. In some cases it may be excellent for temporary stoppings.

